# A call for inclusive research, policies, and leadership to close the global women’s health gap

**DOI:** 10.1186/s13293-024-00669-1

**Published:** 2024-11-08

**Authors:** Irene O. Aninye

**Affiliations:** Society for Women’s Health Research, Washington, DC 20036 USA

**Keywords:** Global health, Policy, Sustainable development goals, Women’s health

## Abstract

Women comprise approximately half of the world’s population, yet they are often underrepresented and inadequately considered in medical and public health research and in health care delivery in the United States and around the world. Elucidating sex and gender differences in disease and fundamental hormonal drivers of women’s health is instrumental to informing our overall understanding of human health and improving women’s health outcomes across the lifespan. The Society for Women’s Health Research and ECH Alliance–The Global Health Connector hosted a women’s health program as part of the United Nations 79th General Assembly Science Summit. Here, I briefly describe the basis for this convening to address global gender health gaps and reflect on the event’s presentations and discussions to recognize and better integrate women’s unique health needs in the sustainable development goals.

## Highlights

The health of women is undervalued and under-researched across the globe. Investing in women’s health benefits everyone. When we improve women’s health, we improve health care for all.

Prioritizing women’s health ensures that the positive outcomes from research advancements and health care interventions can be sustained during the pursuit of other global development goals.

It is critical that stakeholders connect and collaborate with partners to develop the necessary evidence base to enhance individual efforts and dissemination of resources.

Women must be represented in leadership, not only for women’s health initiatives, but also in broader research, health care, and policy decision-making to ensure diverse and inclusive perspectives that produce best practices and outcomes.

Women face unique health challenges over the course of their lives, due to a combination of biological sex differences and sociocultural influences related to gender [1]. Disparities exist and have been documented for women across disease states and life stages that touch every race, ethnic group, geographic location, and socioeconomic status. A better understanding of these health gaps hinges on rigorous and reproducible research that is designed with the intent to address sex and gender differences, rather than retrofitted to apply limited data towards women’s health when convenient [2]. Unfortunately, the investment in research on women’s health is grossly inadequate, especially compared to the disease and economic burdens associated with many conditions that disproportionately affect women [3].

**Fig. 1:**
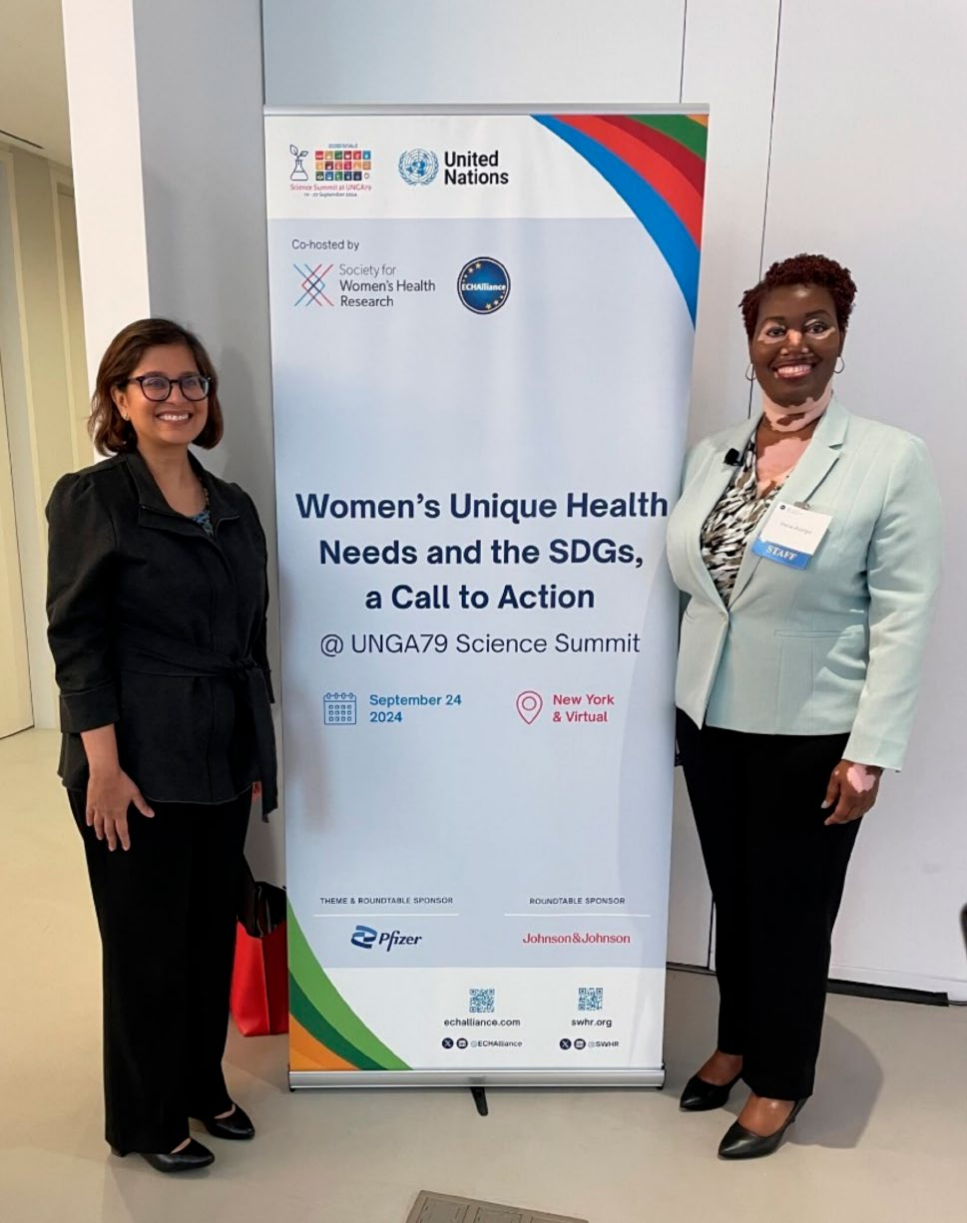
Image courtesy of the Society for Women’s Health Research

On September 24, 2024, leaders across research, health care, government, and industry convened in New York, NY (USA), and virtually across the globe, to elevate conversations and strategies to address global gender health gaps at the United Nations 79th General Assembly (UNGA79) Science Summit. During the Summit, the Society for Women’s Health Research (SWHR) and ECHAlliance-The Global Health Connector co-hosted “Women’s Unique Health Needs and the Sustainable Development Goals,” a series of panels, keynotes, and roundtables that aimed to identify key opportunities to advance women’s health research, care, and policies at both federal and multinational levels. (Fig. 1)

While women’s health is easily associated with certain sustainable development goals, such as good health and wellbeing (Goal 3) and gender equality (Goal 5), attendees were challenged to consider how women’s unique health needs influence and are influenced by other goals, such as zero hunger, quality education, reduced inequalities, and partnerships for the goals (Goals 2, 4, 10, and 17 respectively). SWHR was also encouraged that the United Nations recognized the importance of incorporating women’s health across the lifespan in global health and international policy and when measuring successful implementation and advancement towards One Health [4].

SWHR has long advocated for the national prioritization and integration of women’s health research and care in the United States through federal funding and health care policies that promote science-based interventions and equitable, patient-centered health care. For far too long in the United States – and globally – women have been overlooked and left behind in scientific endeavors. Women have been systematically and systemically excluded from participating in clinical trials, and continue to be underrepresented in science production itself, including in senior academic and leadership positions and health care policy decision-making [5]. Decades after the passage of the National Institutes of Health Revitalization Act of 1993, mandating the appropriate inclusion and reporting of women and underrepresented minorities in clinical research funded by the Institute, we are still working to address these disparities, report data by sex, revise health care interventions, and eliminate the pervasive stigma surrounding women’s health [6] (Fig. 1).

The UNGA79 event shifted women’s health conversations from the often-regarded focus of “bikini medicine” that centers gynecologic and reproductive health to whole body health and women’s health across the lifespan. Women’s unique health needs discussed comprised a diverse spectrum of health conditions, life stages, and diseases that present disparities in women compared to men and among different subpopulations of women, either through incidence or through outcomes. This program brought to a global platform the understanding that women’s health must incorporate all areas of the body to best understand sex and gender differences in health and disease. Takeaways from this meeting suggest that this position is what will improve societal outcomes and ultimately improve women’s health long-term.

Policy solutions offered during the convening emphasized the importance of leveraging opportunities to advance women’s health care and research that focuses on disparities in health conditions that disproportionately affect women. Solutions also addressed barriers to accessing preventive care and medical interventions to improve health outcomes for women and the role of women in health care policy and decision-making, including representation and leadership in the health care workforce at all levels.

Throughout the day, speakers echoed the need for further funding, more visibility, and additional education on women’s health research for everyone – from women and the general public to health care providers and policymakers. From SWHR’s historical perspective and current work, particularly following this global conversation at UNGA79, I challenge the scientific community to reframe how it thinks about sex differences research and women’s health. A broader approach to research and policy programs – one that includes goals to close the gender health gap – will ultimately elevate all human health, and position women for more optimal health and wellness.

Further, I encourage all health care stakeholders to appreciate the value of investing in women’s health research and commit to supporting women’s health across the research continuum. These changes will add up. The UNGA79 conversations have proven that standing up and leading the way for women’s health in each of our spheres of influence is the only way to successfully and sustainably close global gender health gaps – from bench to beside to bureaucracy.

## Data Availability

Not applicable.

## Abbreviations

SWHR: Society for Women’s Health Research
UNGA79: United Nations 79th General Assembly
